# SOX10 regulates multiple genes to direct eumelanin versus pheomelanin production in domestic rock pigeon

**DOI:** 10.1111/pcmr.12778

**Published:** 2019-03-21

**Authors:** Eric T. Domyan, Jeremy Hardy, Tanner Wright, Cody Frazer, Jordan Daniels, Joshua Kirkpatrick, Jacob Kirkpatrick, Kazumasa Wakamatsu, Jonathon T. Hill

**Affiliations:** ^1^ Department of Biology Utah Valley University Orem Utah; ^2^ Department of Chemistry Fujita Health University School of Health Sciences Toyoake Japan; ^3^ Department of Physiology and Developmental Biology Brigham Young University Provo Utah

**Keywords:** differential gene expression, melanogenesis, pigeon, RNA‐seq, Sox10

## Abstract

The domesticated rock pigeon (*Columba livia*) has been bred for hundreds of years to display an immense variety of ornamental attributes such as feather color and color patterns. Color is influenced by multiple loci that impact the type and amount of melanin deposited on the feathers. Pigeons homozygous for the “recessive red” mutation, which causes downregulation of *Sox10,* display brilliant red feathers instead of blue/black feathers. *Sox10* encodes a transcription factor important for melanocyte differentiation and function, but the genes that mediate its promotion of black versus red pigment are unknown. Here, we present a transcriptomic comparison of regenerating feathers from wild‐type and recessive red pigeons to identify candidate SOX10 targets. Our results identify both known and novel targets, including many genes not previously implicated in pigmentation. These data highlight the value of using novel, emerging model organisms to gain insight into the genetic basis of pigment variation.

**Significance**

The tremendous amount of variation in domesticated organisms provides a unique opportunity to understand the genetic basis of phenotypic evolution. However, most studies of variation in non‐model organisms limit their investigation to identifying the causative loci, without probing the biological mechanisms leading to variation. This study utilizes a mutation in pigeons that affects expression of an important melanocyte transcription factor as a novel method for investigating its gene regulatory targets. Comparing the targets between birds and mammals gives us a better understanding of melanocyte biology in general and how pigmentation is controlled in different organisms.

## 1 INTRODUCTION

Color variation is a characteristic of intense scientific interest, because of its visual appeal and frequency as a target in both natural and sexual selection. Among vertebrates, a broad assortment of colors has evolved to provide camouflage, attract mates, and in the case of domestic animals, to suit breeder preferences. In mammals and many birds, the various shades of color are due to differences in the absolute and relative amounts of two types of melanin: eumelanin (brown/black) and pheomelanin (yellow/red) (Serra, 1946). Although eumelanin and pheomelanin have different chemical compositions and structures, they are both synthesized from the same precursor molecule, tyrosine (Hearing et al., 1992), by the enzymes Tyrosinase (TYR, encoded by *Tyr)* and its paralogs Tyrosinase‐related proteins 1 and 2 (encoded by *Tyrp1 *and *Tyrp2*, respectively). Mammalian studies suggest that tyrosine is efficiently converted into eumelanin under high levels of TYR expression, while under lower levels, tyrosine is instead converted into pheomelanin, indicating that TYR expression levels may be directly involved in regulating coat color. TYRP1 and TYRP2 also catalyze important steps in eumelanin biosynthesis but are dispensable for pheomelanin production.

Much of what we understand regarding the regulation of *Tyr* expression, and therefore the switch between eumelanin versus pheomelanin biosynthesis, comes from studies in mouse and other mammalian models (Jackson, 1997; Le Pape, Wakamatsu, Ito, Wolber, & Hearing, 2008). Within the hair follicle, the amount of *Tyr* expression is largely effected by *Microphthalmia‐associated transcription factor* (*Mitf)*, which is in turn regulated by the level of cAMP and protein kinase A signaling induced by the transmembrane Melanocortin‐1 receptor (MC1R) (Walker & Gunn, 2010). MC1R signaling is promoted by the agonist α‐Melanocyte Stimulating Hormone (α‐MSH, encoded by *Pomc*) and inhibited by the antagonist Agouti signaling peptide (ASIP, encoded by *Agouti*). In addition, protein kinase C signaling promotes eumelanin synthesis via stimulation of Tyrosinase activity (Park, Perez, Laursen, Hara, & Gilchrest, 1999; Park, Russakovsky, Ohno, & Gilchrest, 1993). One of the few genes that appears to directly promote pheomelanin synthesis is *Slc7a11, *encoding a cysteine transporter (Chintala et al., 2005). In both mice and humans, the majority of genetic variants that alter the ratio of eumelanin‐to‐pheomelanin production are found in *Mc1r *or *Agouti, *although rare mutations in *Pomc *have been documented in humans (Krude et al., 1998). Mutations in these genes have also been implicated in color variation in other vertebrates (Cal, Suarez‐Bregua, Cerdá‐Reverter, Braasch, & Rotllant, 2017; Guernsey et al., 2013; Yu et al., 2013).

**Figure 1:**
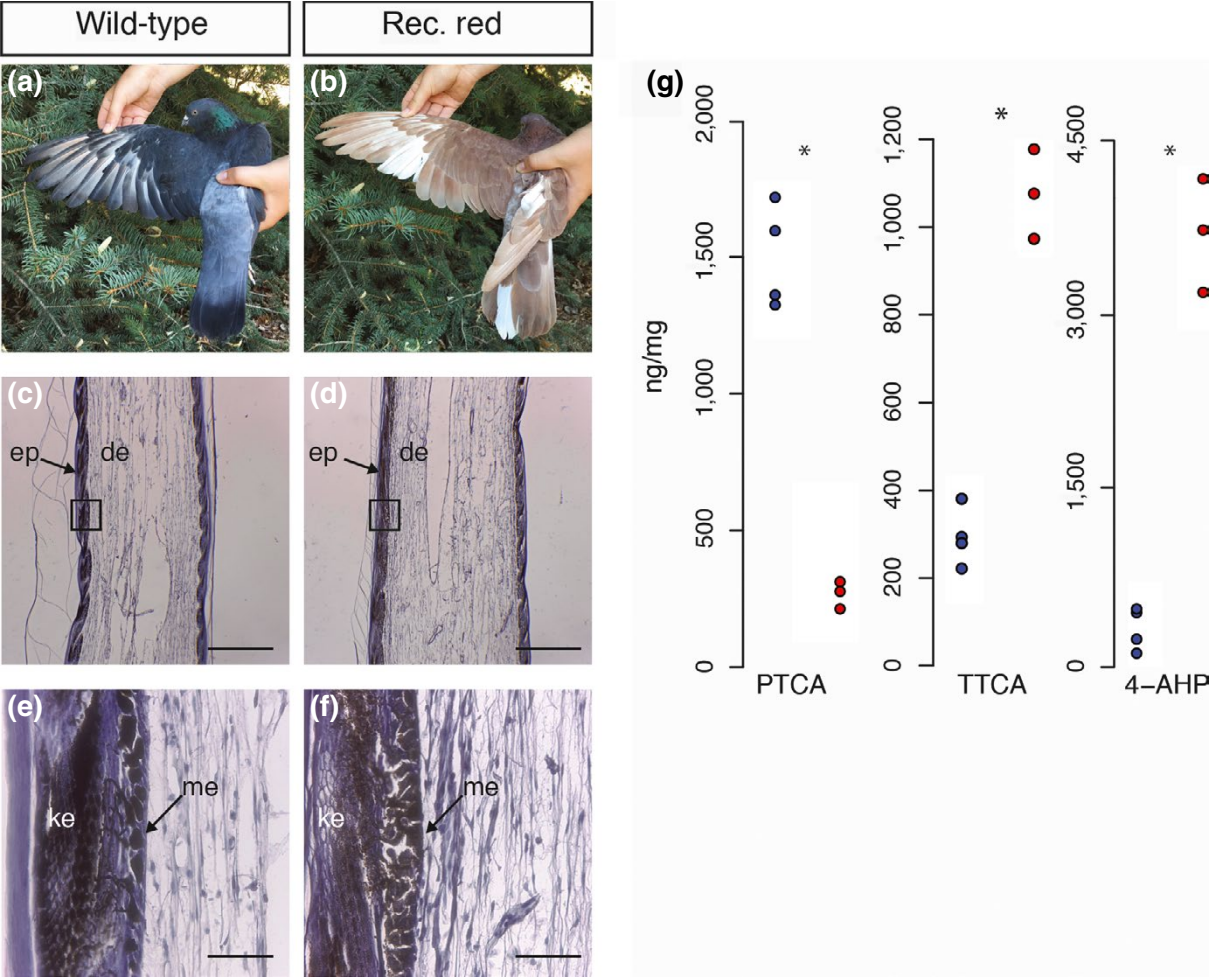
Characterization of wild‐type and recessive red phenotypes.** (**a‐b) Photographs of typical wild‐type (a) and recessive red (b) pigeons. (c‐f). Hematoxylin‐stained section of a regenerating feather from a wild‐type (c,e) and recessive red (d,f) pigeon. Boxed areas in (c,d) are shown in (e,f), respectively. Scale bar in (c,d) 500 µm. Scale bar in (e,f) 50 µm. de: dermis; ep: epidermis; ke: keratinocytes; me: melanocytes. (g). Quantification of eumelanin marker PTCA and pheomelanin markers TTCA and 4‐AHP in wild‐type (blue) and recessive red (red) feathers. Each circle represents a sample from an individual bird. PTCA: wt = 1,499 ± 189 ng/mg, recessive red = 269 ± 50 ng/mg, *p = *0.05; TTCA: wt = 293 ± 66 ng/mg, recessive red = 1,077 ± 102 ng/mg, *p = *0.05; 4‐AHP: wt = 326 ± 181 ng/mg, recessive red = 3,699 ± 485 ng/mg, *p = *0.05. *p = 0.05

Domestic pigeon breeds represent a remarkable repository of phenotypic diversity and were even proposed by Charles Darwin as a particularly useful model for understanding evolution under selection (Darwin, 1859,1868). As a result of intense artificial selection, breeders and hobbyists have generated a tremendous variety of feather colors within this species (Levi, 1965,1969). Importantly, they share the same basic mechanism of pigmentation with mammals (Ito & Wakamatsu, 2003). While the Mendelian patterns of inheritance of many feather colors and patterns in pigeons have been studied, only a few mutations have been characterized at the molecular level (Domyan et al., 2014; Vickrey et al., 2018). One phenotype, dubbed “recessive red” by pigeon hobbyists, causes a uniform red feather color due to high pheomelanin and low eumelanin levels (Figure 1a,b) (Haase, Ito, Sell, & Wakamatsu, 1992). Genomic comparisons between wild‐type and recessive red pigeons implicate *cis‐*acting mutations upstream (*e^1^* and *e^2^* alleles) of *Sry‐Box 10* (*Sox10*), encoding a transcription factor important for melanocyte development and function, as the causative locus (Domyan et al., 2014). *Sox10 *also appears to play a similar role in another avian species, the domestic chicken (Gunnarsson et al., 2011). Similar to pigeon, downregulation of *Sox10 *causes increased pheomelanin production. This stands in contrast to studies in mouse, in which mutations that promote pheomelanogenesis are largely confined to *Mc1r and Asip.*

SOX10 is known to regulate multiple genes during melanocyte development and function including *Mitf*, *Tyr, Tyrp1, Tyrp2, *and others (Baxter, Moreland, Nguyen, Wolfsberg, & Pavan, 2010; Domyan et al., 2014; Jiao et al., 2004; Ludwig, Rehberg, & Wegner, 2004; Murisier, Guichard, & Beermann, 2006,2007; Potterf, Furumura, Dunn, Arnheiter, & Pavan, 2000; Verastegui, Bille, Ortonne, & Ballotti, 2000; Yokoyama, Takeda, & Shibahara, 2006); however, a full investigation into its function in differentiated melanocytes is hindered by the fact that complete loss of *Sox10* function abrogates melanocyte development entirely (Antonellis et al., 2006). Previous research has attempted to identify SOX10 targets by comparing gene expression between wild‐type and *Sox10* ^+/−^ mouse melanocytes in vitro (Fufa et al., 2015). An alternative approach is to use the recessive red phenotype in pigeons. In this study, we perform differential gene expression analyses between wild‐type and recessive red pigeons to identify putative SOX10 targets and perform DNA‐binding motif analyses to identify which targets are likely to be directly regulated by SOX10 binding. Furthermore, we compare our results to those generated in mouse to determine how similar the effects of *Sox10 *downregulation are between avian and mammalian models. Together, these data provide a more‐comprehensive picture of the role of SOX10 during pigment production in pigeon and why its downregulation leads to production of pheomelanin instead of eumelanin.

## 2 METHODS

### 2.1 Animals used

For melanin quantification and differential gene expression analyses via RNA‐seq, four phenotypically wild‐type and four phenotypically recessive red pigeons were initially selected from a local hobbyist's flock. For both phenotypes, two males and two females were selected. A small blood sample was taken for DNA extraction, and birds were genotyped for the recessive red mutation as well as two hypostatic loci (Levi, 1969); the major color locus (*B; *coding variants in *Tyrp1*) and the check locus (*C; *regulatory variants in *Ndp*) (Domyan et al., 2014; Vickrey et al., 2018). *Tyrp1 *expression is abrogated in recessive red birds (Domyan et al., 2014), while the mechanism causing recessive red's epistasis over *Ndp *is unknown. One of the birds initially included in the study as a recessive red pigeon was later found to have been improperly genotyped and was excluded from the analyses, leaving a total of four wild‐type (*E^+^E^+^*) and three recessive red (*e^2^e^2^*) (Domyan et al., 2014) pigeons (Supporting Information Table S1).

For qRT‐PCR validation of differentially expressed genes, six pigeons of each genotype were selected from the same hobbyist. Each group contained one pigeon that had been used for RNA‐seq, and five new pigeons that had not been previously utilized. Animal protocols were approved by the Utah Valley University Institutional Animal Care and Use Committee and comply with the National Research Council's Guide for the Care and Use of Laboratory Animals.

### 2.2 Histology of regenerating feathers

To stimulate feather regeneration, adult secondary flight feathers were plucked and allowed to regrow for 8 days. Regenerating pigmented feathers were plucked and fixed in 4% PFA at 4°C overnight. Feathers were then dehydrated in ethanol, cleared in xylenes, and embedded in paraffin. Sections were cut at 7 µm thickness and stained with hematoxylin using a standard protocol. After hematoxylin staining, the melanin in wild‐type and recessive red tissues was markedly darkened. Eosin was omitted from the staining protocol, as it interfered with visualization of the melanin.

### 2.3 Chemical analysis of feather melanins

Adult feather samples were homogenized in water with a Ten‐Broeck homogenizer at a concentration of 10 mg/ml, and 100 µl aliquots were subjected to alkaline hydrogen peroxide oxidation to yield the eumelanin marker pyrrole‐2,3,5‐tricarboxylic acid (PTCA) and the pheomelanin marker, thiazole‐2,4,5‐tricarboxylic acid (TTCA) (Ito et al., 2011), and hydroiodic acid hydrolysis to yield the pheomelanin marker, 4‐amino‐3‐hydroxyphenylalanine (4‐AHP) (Wakamatsu, Ito, & Rees, 2002), and analyzed by HPLC. Amounts of each marker are reported as ng of marker per mg of feather tissue and compared using Wilcox‐test. Differences were considered statistically significant if *p* ≤ 0.05*.*

### 2.4 RNA Extraction and Sequencing

To harvest RNA from melanocytes, several secondary flight feathers were plucked from one wing of each pigeon to stimulate regeneration and allowed to regrow for 9–10 days. 70% ethanol was used to sterilize as well as to dampen the feathers in the area to assist in locating the regenerating feathers. Once located, each pigmented regenerating feather was plucked, and the proximal 4–5 mm was dissected, bisected longitudinally, and placed in RNA Later (Qiagen, Germantown, MD, USA) overnight at 4°C. Afterward, the dermal pulp was discarded and the feather collar was harvested and stored at −80°C until RNA extraction with RNeasy Plus Mini Kit (Qiagen). RNA integrity was assessed using a TapeStation (Agilent Genomics, Santa Clara, CA, USA), and all samples were sequenced using 50 bp, single‐end reads on one lane of an Illumina HiSeq 2500 at the University of Utah High Throughput Genomics Core.

### 2.5 Differential gene expression analysis

Quality of the sequenced reads was examined using FastQC (https://www.bioinformatics.babraham.ac.uk/projects/fastqc/). Sequence reads were aligned to the reference pigeon genome (Holt et al., 2018) using Rsubread (Chen, Lun, & Smyth, 2016), and differentially expressed genes were identified by DESeq2 using default parameters (Love, Huber, & Anders, 2014). Genes which had an FDR ≤ 0.05 were retained as “differentially expressed” between the two phenotypes.

### 2.6 GO Term Enrichment Analysis

To investigate possible functional enrichment of gene sets, Gene Ontology (GO) term enrichment analysis (geneontology.org/page/go‐enrichment‐analysis) using Fisher's exact test with FDR multiple comparison correction was performed, using a gene list derived from *Gallus gallus*. Categories that had an FDR ≤ 0.05 were retained as “enriched.”

### 2.7 qRT‐PCR validation of differentially expressed genes

RNA was harvested from regenerating feathers, and 1µg was reverse transcribed to cDNA using RevertAid First Strand cDNA Synthesis Kit (ThermoFisher, Waltham, MA, USA). Intron‐spanning primers were designed for 11 differentially expressed genes (Supporting Information Table S2) and used for qRT‐PCR using Apex qPCR GREEN Master Mix (Apex Bioresearch Products, El Cajon, CA, USA) on a CFX96 Touch Real‐Time PCR Detection System (Bio‐Rad, Hercules, CA, USA). Melt‐curve analyses were performed to determine specificity of the amplicon. *ß‐actin* expression was used for normalization. Expression levels of recessive red relative to wild‐type pigeons are reported as mean ± *SD* and compared using Wilcox‐test. Differences were considered statistically significant if *p* ≤ 0.005 (*p* ≤ 0.05/11 to account for multiple testing).

### 2.8 SOX10 Binding‐Site Motif Analysis

DNA‐binding motif analyses were performed using TFBStools (Tan & Lenhard, 2016). Genomic regions spanning 2 kb upstream to 1 kb downstream from the transcription start site of each gene that had an absolute value log_2 _fold change ≥1 and FDR ≤ 0.05 between recessive red and wild‐type pigeons were scanned. Genes with at least one site containing a putative binding‐site score >90% of the maximum score obtainable from the SOX10 position weight matrix in the JASPAR database (Khan et al., 2018) were retained as candidates for direct regulation by SOX10.

### 2.9 Cross‐species Comparison of *Sox10* Downregulation

Data investigating the role of *Sox10* in mouse melanogenesis have been published (Fufa et al., 2015). We utilized these data to determine which of the differentially expressed genes in recessive red pigeons were near a genomic locus bound by SOX10 protein in mouse melanocytes and/or differentially expressed in *Sox10* ^+/−^ mouse melanocytes.

## 3 RESULTS

### 3.1 Characterization of the recessive red phenotype

The recessive red phenotype is characterized by brilliant red, rather than blue/black, plumage. To better understand the cellular basis of the phenotype, we collected histological sections of regenerating wild‐type and recessive red feathers. Feathers of both phenotypes were similar in cellular composition, with abundant keratinocytes, melanocytes, and dermal cells (Figure 1c‐f). A previous study demonstrated that the recessive red phenotype was caused by decreased eumelanin and increased pheomelanin (Haase et al., 1992); however, this study was performed before the molecular identity of the recessive red locus was known. We, therefore, sought to verify these results using pigeons that were molecularly genotyped as *e^2^e^2^* mutants. Consistent with the previous study, feathers of recessive red pigeons had lower levels of the eumelanin marker PTCA, and higher levels of pheomelanin markers TTCA and 4‐AHP, relative to wild‐type pigeons (Figure 1g).

### 3.2 Multiple genes are differentially expressed in recessive red versus wild‐type pigeon feathers

Because *Sox10 *encodes a transcription factor, its target genes should be expressed at different levels in the melanocytes of wild‐type versus recessive red (*Sox10* mutant) pigeon feathers. To identify genes that were differentially expressed (DE genes) in recessive red feathers, we sequenced the transcriptomes from regenerating feathers of four wild‐type and three recessive red pigeons using RNA‐seq (Supporting Information Table S3). After quality filtering and alignment to the reference genome, the dataset contained an average 25.1 million mapped reads/sample, ranging from a minimum of 18.9 million to a maximum of 28.9 million mapped reads. On average, 87.5% reads from each sample were mapped, ranging from a minimum of 86.1% to a maximum of 88.5%. We performed GO Term enrichment analysis for Biological Process of the 1,000 most‐highly expressed genes among the wild‐type birds. Consistent with the fact that feather collar cells were harvested for RNA‐seq, “pigment” was an enriched category, (14 genes, 4.32× enrichment, FDR = 0.001), as was “epidermis development” (15 genes, 3.43× enrichment, FDR = 0.005).

**Figure 2:**
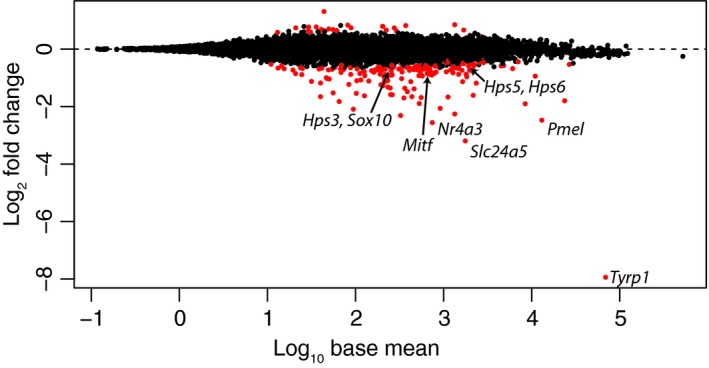
Differential gene expression analysis of wild‐type versus recessive red pigeons. Plot showing log_10 _base mean expression (*x*‐axis) versus log_2 _fold change (*y*‐axis). Genes with FDR ≤ 0.05 in red. Differentially expressed pigment genes indicated by name

Next, we performed differential gene expression analyses to identify which genes were expressed at different levels between recessive red and wild‐type pigeons. We identified 186 downregulated and 25 upregulated genes (FDR ≤ 0.05). (Figure 2, Supporting Information Tables S3, S4). Five of the nine genes with the most severe levels of downregulation, *Tyrp1, Slc24a5, Nr4a3, Pmel,* and *Gsta2 *(LOC102092657 in the current genome annotation), have been implicated in pigment production. *Tyrp1 *encodes an enzyme directly involved in eumelanin synthesis, is a previously identified target of SOX10 in mouse and pigeon, and mutant alleles are associated with the synthesis of brown, rather than black, eumelanin (Bennett, Huszar, Laipis, Jaenisch, & Jackson, 1990; Domyan et al., 2014; Lyons, Foe, Rah, & Grahn, 2005; Murisier, Guichard, & Beermann, 2006). *Slc24a5 *encodes a cation exchanger that localizes to the melanosome membrane, and mutant alleles are associated with reduced pigmentation (Lamason et al., 2005). *Nr4a3 *is induced by MC1R signaling, downregulated by ASIP, and encodes a nuclear receptor that has been linked to premature graying in horses when overexpressed (Le Pape et al., 2009; Smith et al., 2008; Jiang et al., 2012). *Pmel *encodes a melanosome matrix protein, and mutant alleles are associated with reduced pigmentation (Kobayashi et al., 1994; Rinchik et al., 1993). *Gsta2 *is downregulated by ASIP, and encodes a glutathione‐S‐transferase that may influence pheomelanin production through modulation of thiol levels (Granholm, Reese, & Granholm, 1996; Le Pape et al., 2009). Thus, these data suggest the recessive red phenotype may result from changes in multiple pigmentation genes reducing overall pigmentation and converting melanogenesis to pheomelanin production.

GO term enrichment analyses for biological process identified “pigmentation” and several related subclasses as the only significantly enriched categories among downregulated genes, with 8 differentially expressed genes with this annotation (*Hps3, Hps5, Hps6, Mitf, Pmel, Slc24a5, Sox10, and Tyrp1) *(*p = *6.57 × 10^‐3^). Two additional downregulated genes, *Nr4a3 *and *Melan‐A, *also play roles in pigmentation (Aydin, Beermann, Hummler, & Smit, 2012; Smith et al., 2008; Jiang et al., 2012), although they did not include this GO term in their annotation. In contrast, no significant enrichment for biological process was detected among upregulated genes (data not shown).

The majority of pigment genes were detected at similar levels between wild‐type and recessive red pigeons, including those implicated in pigment‐type switching such as *Tyr,* *Mc1r, Slc7a11, Asip*, and *Pomc *(Supporting Information Table S3)*.* The dermal marker gene *Twist2* (Li, Cserjesi, & Olson, 1995) and the 15 most‐highly expressed genes with the GO term “epidermal development” (data not shown) were also expressed similarly between phenotypes. These data suggest the cellular compositions of the feather samples did not significantly differ by phenotype, and therefore the set of DE genes reflect legitimate differences in gene expression.

**Figure 3:**
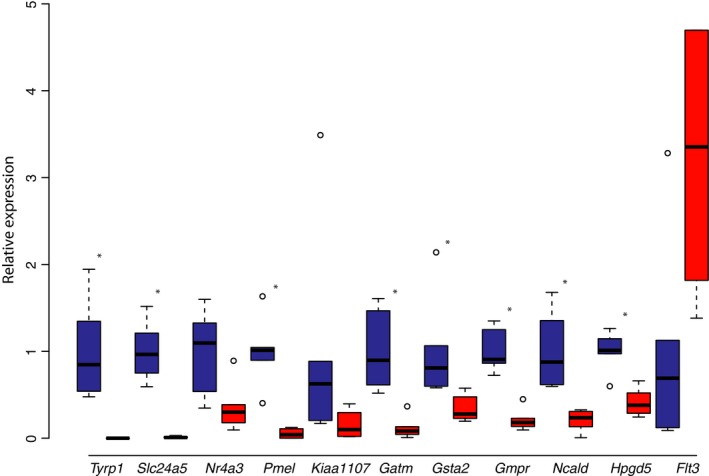
qRT‐PCR validation of differentially expressed genes identified by RNA‐seq. Gene expression comparisons between wild‐type (blue) and recessive red (red) pigeons. Boxes span first to third quartiles; black line, median. *Tyrp1 *relative expression: wt = 1 ± 0.56, recessive red = 0.00027 ± 0.00028, *p = *0.002; *Slc24a5 *relative expression: wt = 1 ± 0.35, recessive red = 0.011 ± 0.01, *p* = 0.002; *Nr4a3 *relative expression: wt = 1 ± 0.49, recessive red = 0.36 ± 0.28, *p* = 0.03; *Pmel *relative expression: wt = 1 ± 0.39, recessive red = 0.052 ± 0.059, *p* = 0.004; *Kiaa1107 *relative expression: wt = 1 ± 1.25, recessive red = 0.16 ± 0.16, *p* = 0.03; *Gatm *relative expression: wt = 1 ± 0.48, recessive red = 0.12 ± 0.13, *p* = 0.002; *Gsta2 *relative expression: wt = 1 ± 0.59, recessive red = 0.34 ± 0.15, *p* = 0.002; *Gmpr *relative expression: 1 ± 0.24, recessive red = 0.21 ± 0.13, *p* = 0.002; *Ncald *relative expression: wt = 1 ± 0.45, recessive red = 0.21 ± 0.12, *p* = 0.002; *Hpgd5 *relative expression: wt = 1 ± 0.22, recessive red = 0.41 ± 0.16, *p* = 0.004; *Flt3 *relative expression: wt = 1 ± 1.18, recessive red = 4.49 ± 4.03, *p* = 0.008; *n* = 6 each. *p <* *0.005

We performed qRT‐PCR to validate a subset of the DE genes identified by RNA‐seq. Of the 11 genes tested, eight had statistically significant differences in expression, and the remaining three displayed a clear trend of differential expression but did not achieve statistical significance after Bonferroni correction for multiple testing (Figure 3). This level of concordance between independent experimental approaches suggests that the DE genes identified by RNA‐seq are a valid set of candidate SOX10 targets.

### 3.3 Correlation between putative SOX10 binding sites and the genomic location of DE genes

The set of DE genes likely includes both direct targets of SOX10 (bound directly by SOX10 protein) and indirect targets (further downstream of SOX10 function). To prioritize which were likely to be direct targets, we selected all genes that had an absolute value log_2_ FC ≥ 1 and FDR ≤ 0.05 for further analysis. In total, 46 genes (45 downregulated and 1 upregulated) met this threshold. For each, we scanned from −2 kb to +1 kb of the transcription start site for putative SOX10 binding sites. All but two had at least one putative SOX10 binding site nearby, while many had two potential binding sites in close proximity, consistent with SOX10 functioning both as a monomer and as a homodimer (Fufa et al., 2015) (Supporting Information Table S5). These data suggest that of the genes we queried, the majority may be direct targets of SOX10.

### 3.4 Many DE genes in recessive red pigeons are near SOX10‐occupied loci in mouse

Although the current lack of reagents in pigeon precludes performing ChIP‐seq to directly identify SOX10 binding sites in the pigeon genome, a comparable study in mouse has been published (Fufa et al., 2015). Of the 192 DE genes in recessive red pigeons for which we could identify a clear ortholog in mouse, 84 (44%) were located near a SOX10 binding site identified by ChIP‐seq in mouse melanocytes (Supporting Information Table S4). These data offer modest support of the binding motif analyses, that many of the DE genes could be direct targets of SOX10.

### 3.5 Differential effects of *Sox10 *reduction in pigeon versus mouse

**Figure 4:**
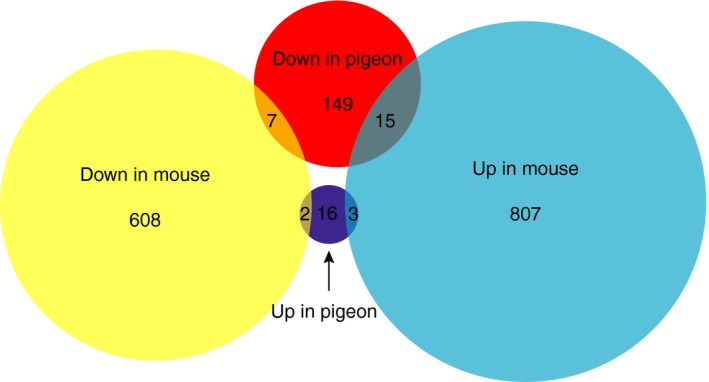
Similarities and differences between differentially expressed genes in recessive red pigeons and *Sox10* ^+/−^ mouse melanocytes. Venn diagram of unique and shared differentially expressed genes. Circle area proportional to number of genes. Red circle = downregulated genes in recessive red pigeon. Dark blue circle = upregulated genes in recessive red pigeon. Yellow circle = downregulated genes in *Sox10* ^+/−^ mouse melanocytes. Light blue circle = upregulated genes in *Sox10* ^+/−^ mouse melanocytes

However, discrepancies became apparent when we compared our set of DE genes in pigeon to DE genes between wild‐type and *Sox10* ^+/−^ mouse melanocytes (Fufa et al., 2015) (Figure 4, Supporting Information Table S4). In contrast to our dataset, which showed a preponderance of downregulated (186) versus upregulated (25) genes in recessive red pigeons, the mouse dataset showed fewer downregulated (614) than upregulated (824) genes. Of the 171 downregulated genes in recessive red pigeon for which a mouse ortholog could be identified, only 7 were also downregulated in mouse, while 15 were upregulated, and the remainder were not differentially expressed. Although “pigmentation” was also an enriched GO term in the mouse dataset, *Pmel *was the only pigmentation gene that was downregulated in both models. Of the three genes encoding the primary melanogenic enzymes (*Tyr, Tyrp1, *and *Tyrp2*), *Tyrp1 *was downregulated only in recessive red pigeons, and *Tyr *and *Tyrp2 *were downregulated only in mouse*. *Among the 21 upregulated genes in recessive red pigeons for which a mouse ortholog could be identified, three were upregulated, two were downregulated, and the remainder were not differentially expressed in mouse. In summary, these results reveal a surprising lack of overlap in DE genes between the two species.

## 4 DISCUSSION

### 4.1 Domestic rock pigeon as a novel model to investigate SOX10 function

The recessive red mutation, causing downregulation of *Sox10*, provides a useful model to investigate the gene regulatory network controlled by this transcription factor during melanogenesis. Indeed, our comparison of gene expression between wild‐type and recessive red pigeons identified both known and novel candidate targets of SOX10. Further, our binding‐site analyses suggest that a majority of the genes we analyzed may be direct targets, while others, such as *Tbx2 *and *Ampd,* are either indirect targets of SOX10, or have SOX10 binding sites outside of the genomic regions queried in our analyses.

### 4.2 *Sox10 *and the promotion of eumelanin over pheomelanin synthesis

Our ultimate goal was to determine how downregulation of *Sox10 *in recessive red pigeons leads to the production of pheomelanin instead of eumelanin. Models suggest that modulation of *Tyr* expression is the primary factor regulating the switch between pigment types (Herraiz, Garcia‐Borron, Jiménez‐Cervantes, & Olivares, 2017). Somewhat surprisingly, neither the current data nor previous examinations identified any differences in *Tyr *expression between wild‐type and recessive red pigeons (Domyan et al., 2014). While pigmentation genes were an enriched category of downregulated genes, none of the DE genes have been previously implicated in regulating eumelanin versus pheomelanin synthesis specifically. Several hypotheses exist to explain these findings. One is that the main mediator (or mediators) of SOX10 promotion of eumelanin over pheomelanin synthesis are not currently implicated in pigmentation. At first glance, this seems unlikely, given the large number of genes known to regulate pigment production (Hubbard, Uy, Hauber, Hoekstra, & Safran, 2010). However, many of these genes were identified through forward genetic screens, and it remains possible that pleiotropic effects of some mutations may preclude a gene's role in pigmentation from being discovered through this approach. This is illustrated by the fact that mutations in at least two DE genes, *Nr4a3 *and *Ncald*, are recessive embryonic lethal in mouse (DeYoung, Baker, Cado, & Winoto, 2003; Duda, Pertzev, & Sharma, 2012), and even *Sox10 *itself has an earlier mutant phenotype that impairs study of its function in differentiated melanocytes (Antonellis et al., 2006).

A second hypothesis is that the production of pheomelanin in recessive red pigeons results from the cumulative effects of multiple genes important for melanin biosynthesis. Both TYRP1 and PMEL promote the proper trafficking and stability of TYR (Kobayashi & Hearing, 2007; Manga et al., 2000; Solano et al., 2000), and although mutations in either individually decreases eumelanin without increasing pheomelanin production (Hirobe, Ito, & Wakamatsu, 2011; Hirobe, Ito, Wakamatsu, Kawa, & Abe, 2014), perhaps their simultaneous downregulation reduces the level of TYR activity enough to promote pheomelanin production.

Because most of our understanding of melanogenesis comes from mammalian models, a third hypothesis is that there are lineage‐specific differences in gene function between mammalian and avian systems, which impairs our ability to nominate a clear candidate mediator of SOX10 promotion of eumelanin. While melanin biosynthesis is thought to be largely conserved among taxa, there is evidence for divergence as well; TYR function may differ slightly between human and mouse, for instance (Olivares, Jiménez‐Cervantes, Lozano, Solano, & García‐Borrón, 2001). These hypotheses are not mutually exclusive, and identifying the best model to explain the relationship between gene expression changes and pheomelanism of recessive red pigeons is an important avenue of future research.

### 4.3 Evolution of SOX10 function in vertebrate pigmentation

A surprising finding was that although the set of DE genes in recessive red pigeons had support from independent experimental approaches, there were a surprisingly small number of DE genes in common between recessive red pigeons and *Sox10* ^+/−^ mouse melanocytes. This could be due to differences in experimental design; the current study utilized an in vivo system of regenerating feathers, while the earlier study utilized mouse melanocytes in vitro (Fufa et al., 2015). A second possibility is that differences in the amount and timing of *Sox10 *reduction in the two models may produce different results. A third possibility, and perhaps the most intriguing, is that *Sox10 *has evolved to play similar yet unique roles during melanogenesis in mouse versus pigeon. Consistent with this hypothesis, partially overlapping *Sox10 *enhancer deletions exist in pigeon, chicken, and mouse and are associated with different phenotypic consequences (Antonellis et al., 2006; Domyan et al., 2014; Gunnarsson et al., 2011). In chicken and pigeon, these deletions are associated with pheomelanin production, but in mouse mutants, the melanocyte lineage is lost. These deletions are not identical, and it therefore, remains possible that differences in their precise breakpoints cause the different phenotypes. However, the hypothesis that SOX10 function has diverged during vertebrate evolution has support in the published literature. In mouse, both MITF and SOX10 are necessary for expression of *Tyr*, but in zebrafish, MITF is sufficient (Hou, Arnheiter, & Pavan, 2006). In short, divergence in SOX10 targets among taxa may allow it to play different roles in different lineages and thus produce different phenotypes when perturbed. As described earlier, these hypotheses are not mutually exclusive or exhaustive, and testing them is an important direction of future research.

## CONFLICT OF INTEREST

The authors declare no conflicts of interest.
